# Trace analysis of multi-class pesticide residues in Chinese medicinal health wines using gas chromatography with electron capture detection

**DOI:** 10.1038/srep21558

**Published:** 2016-02-17

**Authors:** Wei-Jun Kong, Qiu-Tao Liu, Dan-Dan Kong, Qian-Zhen Liu, Xin-Ping Ma, Mei-Hua Yang

**Affiliations:** 1 Institute of Medicinal Plant Development, Chinese Academy of Medical Sciences & Peking Union Medical College, Beijing, 100193, PR. China; 4 Department of Traditional Chinese Medicine, Air Force General Hospital, PLA, Beijing, 100142, PR. China

## Abstract

A method is described for multi-residue, high-throughput determination of trace levels of 22 organochlorine pesticides (OCPs) and 5 pyrethroid pesticides (PYPs) in Chinese medicinal (CM) health wines using a QuEChERS (quick, easy, cheap, effective, rugged, and safe) based extraction method and gas chromatography-electron capture detection (GC-ECD). Several parameters were optimized to improve preparation and separation time while still maintaining high sensitivity. Validation tests of spiked samples showed good linearities for 27 pesticides (*R* = 0.9909–0.9996) over wide concentration ranges. Limits of detection (LODs) and quantification (LOQs) were measured at ng/L levels, 0.06–2 ng/L and 0.2–6 ng/L for OCPs and 0.02–3 ng/L and 0.06–7 ng/L for PYPs, respectively. Inter- and intra-day precision tests showed variations of 0.65–9.89% for OCPs and 0.98–13.99% for PYPs, respectively. Average recoveries were in the range of 47.74–120.31%, with relative standard deviations below 20%. The developed method was then applied to analyze 80 CM wine samples. Beta-BHC (Benzene hexachloride) was the most frequently detected pesticide at concentration levels of 5.67–31.55 mg/L, followed by delta-BHC, trans-chlordane, gamma-BHC, and alpha-BHC. The validated method is simple and economical, with adequate sensitivity for trace levels of multi-class pesticides. It could be adopted by laboratories for this and other types of complex matrices analysis.

Chinese medicinal (CM) health wines have gained wide acceptance due to their high nutritive and medicinal values, unique flavor, and other health functions^1^,^2^, and are frequently consumed in Asia, Europe, and other countries. In particular, these wines are playing an ever increasing role in Asian life, culture, and diet, and consumers have strict requirements concerning their quality.

CM health wines are composed of various Chinese medicinal materials (CMMs) which are subject to insect pests or microbial contaminants during the growing process^3^. A wide range of pesticides are frequently applied during CMMs growth to control pests and increase yield and quality. However, if these harmful chemicals do not degrade naturally or are not completely removed, they are able to penetrate CMM plant tissues^4^,^5^. Even at low or ultra-low levels, upon transfer into CM health wines the pesticide residues are potential risk to human health, as they are particularly persistent and have a tendency for bio-accumulation^6^,^7^. At present, maximum residue levels (MRLs) for pesticides, including those used for CMMs growth have been established in some countries^8^,^9^. These limits, however, have not been applied to processed products, including CM health wines^7^,^10^. Consequently, it is currently mandatory to monitor pesticide residues in CM health wines for consumer protection, compliance with some limited standards, and fair trade certification. The large number and volume of pesticides, as well as low MRLs, necessitates highly sensitive and selective sensing methods.

Because CM health wines originate from medicinal plants and herbal materials, they represent very complicated matrices for analysis of pesticide residues. Furthermore, pesticides usually exist at trace (ppm) or lower levels, making the development of analytical methods and highly sensitive instrumentation a great challenge. In recent years, numerous analytical methods have been reported for screening low-levels of pesticide residues in CMMs and wines^3^,^4^,^11^. Among these, the most commonly used are chromatographic methods, such as liquid chromatography (LC) or gas chromatography (GC) coupled with a range of detectors^3^,^4^,^7^,^10^,^11^,^12^,^13^,^14^. Although gas chromatography coupled to (tandem) mass spectrometry (GC-MS or GC-MS/MS) has improved the detection procedure, unavoidable matrix effects during the extraction process often interfere with detection of target pesticides, making quantification and analysis of residues erroneous and ambiguous. Due to the wide differences in physical and chemical properties, GC coupled with an electron capture detector (GC-ECD) for organchlorine pesticides (OCPs) and pyrethroid pesticides (PYPs), and with a nitrogen phosphorous detector (GC-NPD) for phosphate pesticides, has been widely used and is officially listed in the Chinese Pharmacopoeia^15^.

As a rule, pesticide residue analysis in complex matrices is performed using multi-step methods of extraction and pre-concentration, meaning that sample preparation is a crucial step. A variety of classical sample preparation methods, such as solid phase extraction (SPE)^16^,^17^,^18^, liquid-liquid extraction (LLE)^19^, solid-phase micro-extraction (SPME)^20^,^21^, membrane assisted solvent extraction (MASE)^22^,^23^, along with several modern methods including stir bar sorptive extraction (SBSE)^24^,^25^, and single-drop micro-extraction (SDME)^26^,^27^, etc. have been used to analyze pesticides in complex matrices including wines. However, these methods are usually onerous, requiring large sample volumes, special sorbents, and multiple cleanup steps. In recent decades, extensive efforts have been focused on developing new sample preparation techniques that save time, labor, and solvent consumption with the goal of limiting small matrix interferences and thereby improving overall analytical performance. QuEChERS (quick, easy, cheap, effective, rugged and safe) has been accepted as the “gold standard” for analysis of pesticide residues in various sample matrices^28^,^29^,^30^. Several previous studies have reported on the application of QuEChERS-based methods for analysis of multi-pesticide residues in wines^31^,^32^,^33^. To the best of our knowledge, however, there is not been a previous report on the use of a QuEChERS-based sample preparation procedure for the extraction of pesticides from CM health wines. Furthermore, no analytical method has been outlined for trace determination of multi-pesticide residues with regards to OCPs and PYPs in CM health wines.

The primary objective of this work was to develop and validate a simple, fast, sensitive, and reliable analytical method based on a QuEChERS extraction procedure for the simultaneous identification and quantification of 27 pesticides, including 22 OCPs and 5 PYPs, using GC-ECD. Special attention was paid to optimizing the QuEChERS procedure and the GC-ECD conditions. In addition, an extensive validation study was used to evaluate analytical performance. The developed method was then assessed for its real-world applicability by analyzing multi-pesticide residues in 80 CM health wine samples collected from various Chinese markets.

## Results and Discussion

### Selection of sample preparation technique

Sample preparation is often the most critical issue of any method that deals with multiple residues because of the wide range of polarities, water solubilities, and volatilities of the pesticides that must be simultaneously extracted from the matrix. Co-extraction of interfering substances may lead to low recoveries of trace level pesticides, and should be avoided for efficient extraction. In the present study, two simple and common methods, namely QuEChERS and SPE, were compared for their efficacy in extracting pesticides from CM health wine samples. Results from repeated trials demonstrated that the peak numbers and intensities, together with the extraction efficacy, necessary cleanup and pesticide recovery rate using an SPE method were not better than the results of a QuEChERS technique (data were shown in Supplementary Figure 1). In addition, large amounts of organic solvents were used during the SPE extraction and cleanup, leading to higher costs and a higher possibility of pollution to the environment. Based on the results, extraction of pesticides from CM health wines was performed using a QuEChERS technique.

### Optimization of a QuEChERS based sample preparation

Several parameters that influenced the performance of QuEChERS, such as the type of sorbent, the organic solvent, sample volume, and extraction time, were investigated in order to obtain the highest possible high recovery rates of the target pesticides.

#### Sorbent selection

Because CM health wines are such complex matrices, one of the most important steps in the optimization of the QuEChERS procedure was to select an appropriate sorbent that could effectively remove interfering substances. Various sorbents were tested, including PSA, GCB, alumina oxide, and florisil, based on the knowledge that each sorbent is useful for specific purposes. After testing, florisil was chosen as a sorbent due to its low cost, easy cleanup, and high recoveries as compared to the other sorbents.

Next, the amount of sorbent needed to effectively remove target pesticides was determined. A large amount of sorbent can effectively purify pesticides in complex matrices, but with low overall recoveries. To determine the optimum sorbent level for purification efficiency, different amounts of florisil (100, 200, 300, 400 and 500 mg) were tested and compared. Results showed that using 400 mg florisil provided satisfactory purification efficiency as well as high recoveries for 27 pesticides in CM health wine samples.

#### Extraction solvent optimization

**Figure 1:**
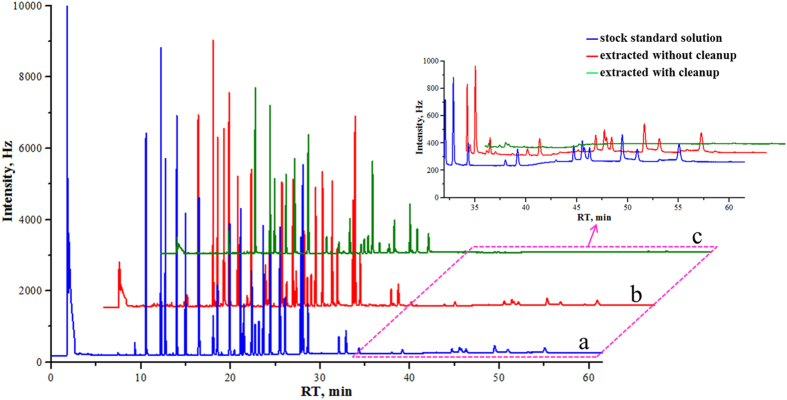
GC-ECD chromatograms of (**a**) standard stock solution, (**b**) fortified sample solution with standards extracted without cleanup, and (**c**) fortified sample solution with standards extracted after cleanup with *n*-hexane as extraction solvent.

**Figure 2:**
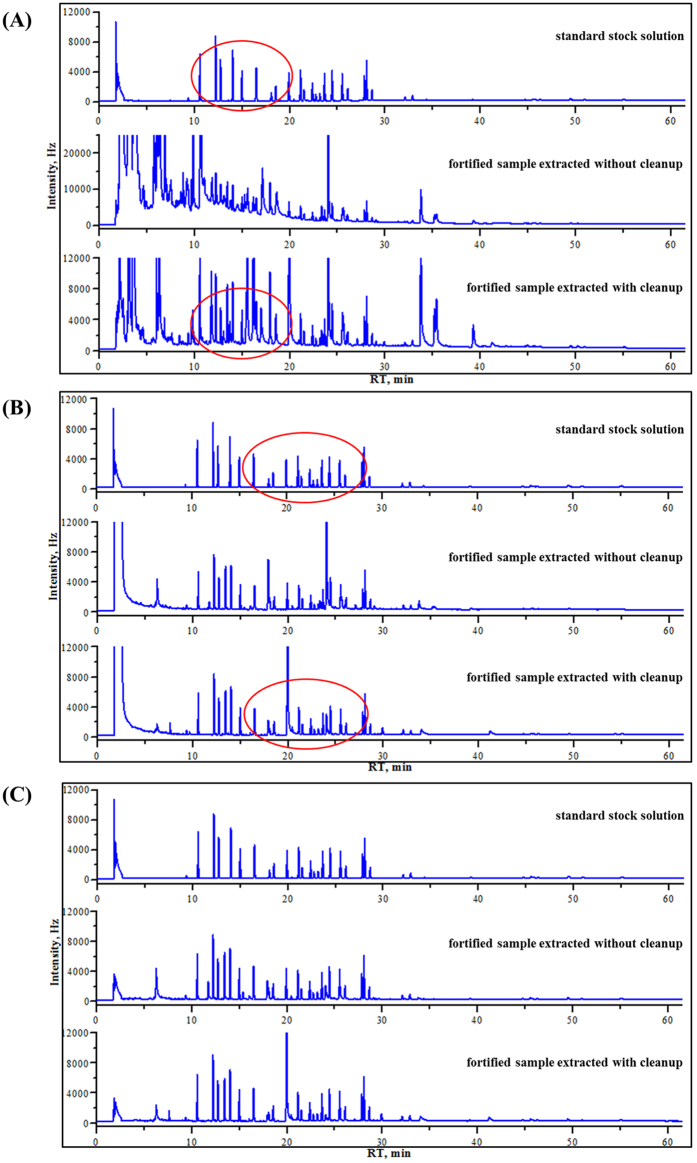
GC-ECD chromatograms of fortified sample solutions with standards extracted with (**A**) ethyl acetate, (**B**) dichloromethane:*n*-hexane (1:1, *v/v*), and (**C**) ethyl acetate:*n*-hexane (1:1, *v/v*) as extraction solvent.

Selection of a suitable extraction solvent is essential for the development of a useful QuEChERS method. In order to achieve high extraction efficiency, organic solvents with different polarities and levels of water solubility were tested and ranked according to their extraction capacity and their overall behavior during GC-ECD analyses. Commonly used solvents with a wide range of polarities, including ethyl acetate, acetone, *n*-hexane, dichloromethane, or mixture of these solvents were tested. Specific mixtures that were tested included dichloromethane with *n*-hexane (1:1, *v/v*), and *n*-hexane with ethyl acetate (1:1, *v/v*). Results showed the following: (1) use of dichloromethane as an extraction solvent led to serious emulsification in the sample; (2) use of *n*-hexane led to a good GC-ECD chromatogram of target analytes (Fig. 1), however some PYP pesticides were adsorbed, leading to low extraction efficacy; (3) in the case of using ethyl acetate (Fig. 2A) or a dichloromethane:*n*-hexane (1:1, *v/v*) mixture (Fig. 2B) as an extraction solvent, precise quantification was difficult because the fortified samples were not satisfactorily purified and interfering peaks were observed simultaneously at the retention times of target pesticides; (4) a mixture of *n*-hexane and ethyl acetate (1:1, *v/v*) gave higher average peak areas along with better overall recovery rates (70–110%) (Fig. 2C), while also exhibiting the lowest relative standard deviation (RSD) values (data not shown) at the retention times of target analytes, and was therefore the preferred extraction solvent.

#### Solvent volume and extraction time optimization

Extraction volume and time both display a positive and significant effect on extraction efficiency of target analytes, and are therefore significant variables to investigate. Raising the volume of the *n*-hexane:ethyl acetate (1:1, *v/v*) solvent from 2.0 to 5.0 mL increased the recovery rates of 27 pesticides, while further increase the volume showed no significant improvement. Further experiments tested extraction times and showed that for the mixture of sample solution and *n*-hexane:ethyl acetate (1:1, *v/v*) solvent, a 2 min extraction procedure provided the highest recovery of all target pesticides in the sample. Based on the results, 5.0 mL of *n*-hexane:ethyl acetate (1:1, *v/v*) solvent and an extraction time of 2 min were chosen for further tests.

### Optimization of GC-ECD conditions

Crucial chromatographic parameters including column type, temperature, flow rate, and injection volume were optimized in order to obtain peak specificity and sensitivity for trace amounts of pesticides in CM health wine samples. The overarching goals were satisfactory separation, sensitive detection, and accurate quantification of trace levels of multiple pesticides.

#### GC capillary column type

Various types of GC columns were tested and an Agilent J & W DB-1701 capillary column, a low/mid-polarity column made of (14%-Cyanopropyl- phenyl)-methylpolysilicone, was chosen as the preferred column because it reduced material loss and provided wonderful separation of the 27 target pesticides.

#### Column temperature

A GC method with a high initial oven temperature can save analysis and recycling time, as well as improve peak-to-peak separation efficiency and peak shapes. However, too high a temperature may lead to evaporation of volatile substances that can both interfere with targets and contaminate of the analytical column and detector. Therefore, different initial oven temperatures and ramping programs were tested. When the initial temperature increased from 90 to 150 °C there was indeed an obvious decrease in the total run time, however for any temperature above 120 °C, the peak widths of the more volatile pesticides increased significantly and expressed poor peak shapes. Based on the above observed facts and the results shown in Supplementary Table 1, 120 °C was chosen as the initial oven temperature with a ramping program rate of 20 °C/min.

#### Flow rate

Another essential parameter influencing the separation efficiency of multiple components in GC analysis is the flow rate of nitrogen. To determine the best flow rate, a series of different constant gas flow rates were tested by evaluating the chromatographic separation and signal-to-noise (S/N) ratio of each target pesticide. The best results were achieved using a flow rate of 1.0 mL/min.

#### Injection volume

Another parameter that needed to be optimized was the injection volume of the samples. Earlier studies have shown that a large injection volume of sample solution generally improves the detection rate in GC analysis^34^,^35^, but too large an injection volume exceeded the tolerable limit of the liner and also raised the pressure. After several tests, all further experiments were completed using an injection volume of 1 μL.

### Method validation

The optimum QuEChERS and GC-ECD conditions described above were used to determine the presence of any quantity target analytes as a way to verify the real world applicability of the method. The validation procedure was performed following the SANCO/10684/2009 European Guidelines^36^. The analytical performance characteristics investigated included selectivity, calibration curve, ability to determine a linear calibration curve, the limit of detection (LOD) and quantification (LOQ), precision, stability, and trueness (expressed as recovery rate). Precision was determined by measuring the relative standard deviation (RSD), and was evaluated as inter- and intra-day precision. In this QuEChERS extraction technique, the volume of the extract phase was smaller than that of the sample, and led to an in-exhaustive extraction of the analytes. Therefore, in this case, pesticides with suitable recoveries (70–120%) were included in the validation process.

#### Selectivity

**Figure 3:**
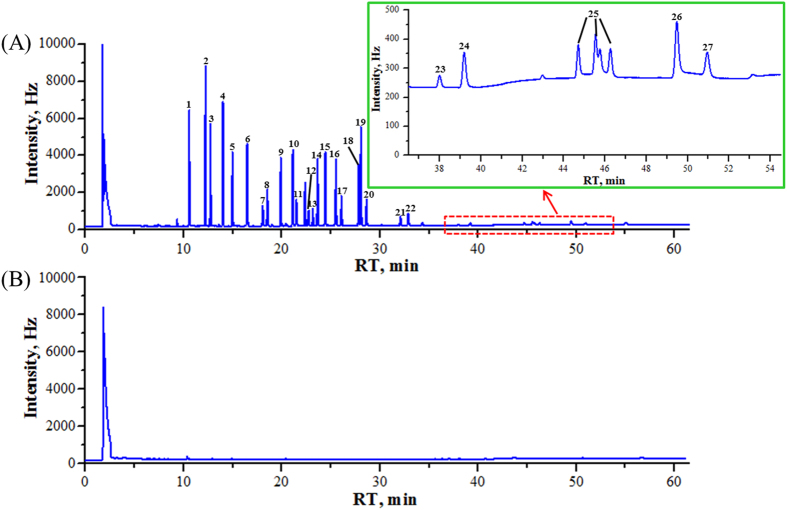
GC-ECD chromatograms of (**A**) fortified CM health wine sample with 27 OCP and PYP standards, and (**B**) control sample. *Peaks* 1. hexachlorobenzene, 2. alpha-BHC, 3. quintozene, 4. gamma-BHC, 5. heptachlor, 6. aldrin, 7. chlorothalonil, 8. beta-BHC, 9. delta-BHC, 10. heptachlor epoxide, 11. triadimefon, 12. alpha-endosulfan, 13. cis-chlordane, 14. trans-chlordane, 15. p.p′-DDE, 16. dieldrin, 17. endrin, 18. o.p′-DDT, 19. p.p′-DDD, 20. beta-endosulfan, 21. p.p′-DDT, 22. methoxychlor, 23. fenpropathrin, 24. permethrin, 25. cypermethrin, 26. flucythrinate, 27. decamethrin.

The selectivity of the proposed method was verified by comparing results from a control (pesticides-free) health wine sample and a sample fortified with 27 pesticides. As was shown in Fig. 3, no interfering peaks were observed at the retention times of individual compounds, proving that there was sufficient selectivity for the analysis of multiple pesticides at trace levels. Figure 3 also showed that the control samples did not give false-positive signals.

#### Calibration curve and linearity

Calibration curves were constructed using standard working solutions at ten concentration levels obtained by diluting the standard stock solution with *n*-hexane (0.001, 0.005, 0.01, 0.05, 0.1, 0.2, 0.4, 0.6, 0.8 and 1.0 ng/mL for 22 OCPs; 0.002, 0.01, 0.02, 0.1, 0.2, 0.4, 0.6, 0.8, 1.0, 2.0 ng/mL for 5 PYPs). These were then analyzed under the chromatographic conditions described previously. An external calibration method based on the peak area of each analyte was then used to quantify targets. A in the form of *y* = A*x* + B was constructed by plotting the peak areas (*y*) against standard concentrations (*x*). The results shown in Table 1 demonstrated that calibration curves with excellent linearity were obtained for the 27 pesticides. The coefficients of determination (*R*) were higher than 0.9900 in a wide concentration range for all targets except permethrin (*R* = 0.9876 in the range of 0.001 ng/mL to 2.0 ng/mL).

#### LOD and LOQ

LODs were estimated using the minimum concentrations detected for all target analytes based on signal-to-noise (S/N) ratio of three and LOQs were set as ten times this ratio. As listed in Table 1, the LOD and LOQ values were found to be at the low ng/L level, with LODs ranging from 0.06 ng/L to 2 ng/L for OCPs and from 0.02 ng/L to 3 ng/L for PYPs, and from 0.2 ng/L to 6 ng/L for OCPs and from 0.06 ng/L to 7 ng/L for PYPs. These values were lower than the MRL of 10 μg/kg or 10000 ng/L established by European Union^8^, and therefore demonstrated that the developed method has sufficient sensitivity for simultaneous determination of multi-pesticide residues at low concentrations.

#### Precision and stability

To gage the precision of the method, intra- and inter-day variations were estimated and expressed as RSD of the signals or peak areas for each analyte following an analysis of 0.1 mg/L standard working solution injected six times consecutively on the same day and injected six times over six consecutive days. The results in Table 1 showed that inter-day variation of peak areas for 27 pesticides were in the range of 0.65–9.89%, and intra-day variations of 0.98–13.99%. These values were in compliance with the requirements of the SANCO document (≤20%). Stability was investigated by injecting a CM health wine sample spiked with 0.5 mg/L of 27 pesticides at 0, 2, 4, 6, 10, and 12 h. The RSD values were lower than 10.15%, as shown in Table 1. All of the above results indicated that the proposed method was precise and that the fortified samples were stable.

#### Trueness

The trueness of the developed method was determined through recovery studies using control samples of CM health wines. Ningxiahong, Chinese jing, and Yedaolugui wines were selected as control samples based on their variety of CM raw materials including raw material from single plant, raw materials from various plants, and raw materials from a mixture of animals and plants, respectively. These samples were fortified at three (high, medium and low) spiking levels, which were 0.5, 0.05, 0.005 mg/kg for OCPs, and 1, 0.1, 0.01 mg/kg for PYPs. Next, all samples were extracted and analyzed in triplicate following the previously described procedure. The recovery percentages were then calculated using the following equation:


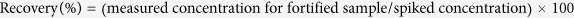


As listed in Table 2, the recoveries for the 27 OCPs and PYPs in the three control samples ranged from 50.38–120.31% for Ningxiahong wine, 47.74–113.65% for Chinese jing wine, and 50.56–110.21% for Yedaolugui wine with RSD values in the range of 0.19–24.69%. These results demonstrated that for nearly all pesticides tested the optimized method achieved recoveries (70–120%) and RSD values (≤20%), in line with criteria set by EU guidelines^36^.

These results demonstrated that the developed method was precise, accurate, and sensitive enough for simultaneous determination of trace levels of 27 pesticide residues in CM health wines.

### Real sample analysis

The validated QuEChERS based extraction procedure coupled with GC-ECD method was then applied to measure levels of 22 OCP and 5 PYP residues in 80 CM health wine samples purchased or collected from various Chinese markets in China. As listed in Table 3, residues of 5 pesticides with contents above the LOQ were detected in 9 samples (11.3%), while the other samples tested negative for pesticide residues. Examples of the positive samples included a home-made ginseng wine that was found to contain 4 pesticides (alpha-BHC, gamma-BHC, beta-BHC and delta-BHC), as well as one Yedaolugui wine and one Jiafang wine that both contained the same 2 pesticides (beta-BHC and trans-chlordane). Out of the detected pesticides, beta-BHC was the most predominant with concentration levels ranging from 5.67–31.55 mg/L, as well as the most common incidence in 8 samples (10%). The next highest concentration was delta-BHC (17.30 mg/L) in 1 sample (1.3%), followed by trans-chlordane (3.58–7.45 mg/L) in 3 samples (3.8%), gamma-BHC (3.83 mg/L) in 1 sample (1.3%), and alpha-BHC (2.48 mg/L) in 1 sample (1.3%). The pesticide concentrations measured using this method were all below the suggested permissible level^8^. It was worth noting that all of the identified residues belonged to OCPs, with no PYPs detected in the tested samples. This may be due to the fact that PYPs have lower stability and are more easily degraded compared to OCPs. In addition, although BHC pesticide has low toxicity, low cost, and is highly effect, it was also the most commonly found OCP in the tested samples due to its chemical stability. The findings of this study reiterate the importance of maintaining strict control of pesticide use and not ignoring the potential harm pesticides may cause to human health. Further work will focus on measuring the pesticide levels in Chinese medicinal raw materials of used to make the wine samples that tested positive in order to better control their contents.

## Conclusion

Analysis of multi-class pesticide residues is a current topic of interest in the field of analytical chemistry. Preparation of samples is often the main constraint for successful analysis. In this study, a simple and rapid method combining a QuEChERS based extraction procedure with GC-ECD was developed and validated for simultaneous monitoring and identifying trace levels of multi-pesticide residues. The QuEChERS procedure had the advantage of combining a simple isolation step for target pesticides with a single step sample clean-up method as compared with other sample preparation procedures^16^,^17^,^18^,^19^. Crucial parameters for QuEChERS extraction and chromatographic analysis were optimized and the developed method was validated. The final method provided a wide concentration range, satisfactory linearity, low LOD and LOQ, good precision, and a high recovery rate, which was comparable with other detection methods for trace levels of pesticides^3^,^11^,^16^,^37^. Next the method was used to simultaneously analyze 22 OCPs and 5 PYPs residues in 80 CM health wine samples. This successful real world application demonstrated that though the validated method used economical, cheap, and simplified extraction and clean-up procedures, it still maintained adequate sensitivity for detection of trace levels of pesticides and could easily be adopted by other laboratories for analysis of complex matrices.

To the best of our knowledge, this is the first report on simultaneous determination of multi-class pesticide residues in CM health wines in China. Total analysis time (less than 70 min, including 10 min for sample preparation plus 60 min for analysis) for 27 pesticides was shorter as compared to traditional methods^3^,^16^,^37^. Therefore high sample throughput could be achieved, and the method could be useful for pesticide monitoring programs that work with a large numbers of samples. This method not only lowered exposure to hazardous and toxic chemicals usually used for wine sample preparation, but also lowered the overall cost of the analysis. This method could therefore be used as a powerful reference for trace-analysis of multi-class contaminants in other complex matrices, including Chinese medicinal raw materials and related products, etc.

## Materials and Methods

### Chemicals and reagents

**Figure 4:**
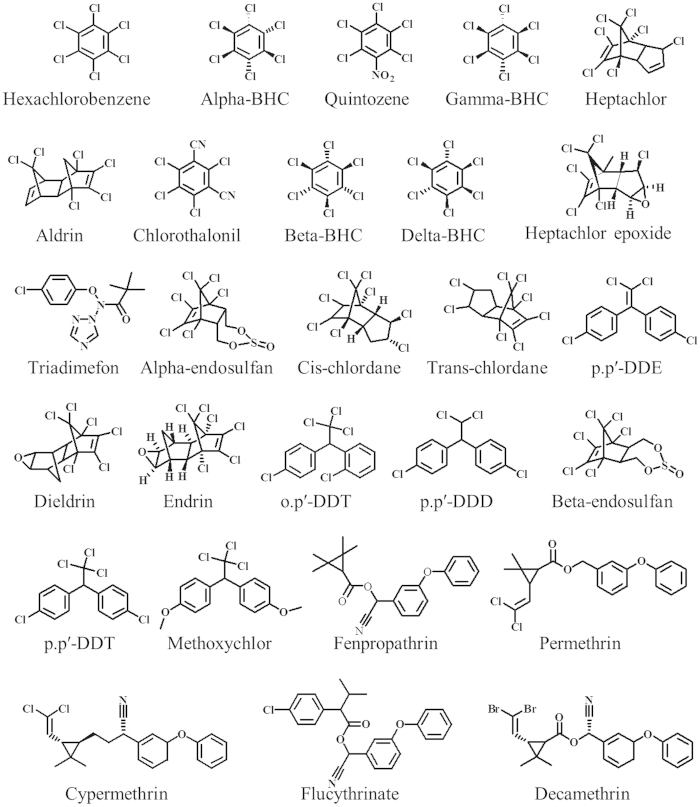
Chemical structures of 27 OCP and PYP pesticides.

Twenty-seven pesticide standards (uncertainty μg/mL) including hexachlorobenzene (±0.05), alpha-BHC (±0.11), quintozene (±0.07), gamma-BHC (±0.25), heptachlor (±0.10), Aldrin (±0.17), chlorothalonil (±0.11), beta-BHC (±0.11), delta-BHC (±0.07), heptachlor epoxide (±0.08), triadimefon (±0.07), alpha-endosulfan (±0.10), cis-chlordane (±0.17), trans-chlordane (±0.11), p.p′-DDE (±0.11), dieldrin (±0.07), endrin (±0.08), o.p′-DDT (±0.10), p.p′-DDD (±0.17), beta-endosulfan (±0.11), p.p′-DDT (±0.11), methoxychlor (±0.07), fenpropathrin (±0.08), permethrin (±0.07), cypermethrin (±0.19), flucythrinate (±0.115), decamethrin (±0.07) were purchased from the Agro-Environment Protection Institute (Tianjin, China). They were stable over a period of at least three months and their chemical structures were shown in Fig. 4.

Ethyl acetate, dichloromethane, n-hexane (HPLC grade) were obtained from Sinopharm Chemical Regent Co., Ltd (Beijing, China). HPLC-grade acetone was from MREDA (IL, USA). Florisil (60–100 mesh) was purchased from Sigma-Aldrich (Bellefonte, PA, USA).

### Instrumentation

All analyses were performed using an Agilent 6890N gas chromatograph (Agilent Technologies, CA, USA) equipped with an ECD detector, an Agilent 7683 autosampler and an injector, connected to an HP ChemStation (Hewlett-Packard, Palo Alto, CA, USA) for instrument control and data analysis. A DB-1701 (30 m × 0.25 mm I.D., 0.25 μm) capillary column was used for chromatographic separation. Injector and detector temperatures were held at 220 °C and 300 °C, respectively. The oven temperature program was set as follows: initial 120 °C held for 1 min, ramped to 170 °C at 20 °C/min for 1 min, followed by ramped to 200 °C at 4 °C/min for 5 min, then ramped to 250 °C at 4 °C/min for 10 min and finally ramped to 270 °C at 10 °C/min for 20 min. The injection volume was 1.0 μL. Total run time was 60 min. Ultra-high purity nitrogen (over 99.99%) was selected as the carrier gas at a constant flow rate of 1.0 mL/min. Injection was performed at splitless mode with a purge time of 0.75 min. Quantification of the pesticides was performed using a external standard method based on the detected and integrated peak area.

### Preparation of standard solution

A stock solution of mixed pesticide standards were prepared in *n*-hexane at the concentration of 100 mg/L and 2 mg/L and stored at −20 °C in a refrigerator. The standard working solutions were daily obtained by appropriate dilution of the stock solution.

### Sampling

A total of 80 CM health wine samples, which could be divided to 25 types, such as Ningxiahong (*n* = 20), Chinese Jing wine (*n* = 14), Lotus white wine (*n* = 5), Yedaolugui wine (*n* = 5), Fenglin wine (*n* = 4), Diyi wine (*n* = 3), Yishebian wine (*n* = 1), Yisheshengbao wine (*n* = 1), Shiguogong wine (*n* = 1), *Cordyceps sinensis* wine (*n* = 1), Sanbian wine (*n* = 2), *Rhodiola rasea* wine (*n* = 1), Ginseng wine (*n* = 1), *Lucid ganoderma* wine (*n* = 1), *Herba saussureae involucratae* wine (*n* = 1), *Tall gastrodia tuber* wine (*n* = 1), Chinese Manoliavine wine (*n* = 1), *Desertliving cistanche* herb wine (*n* = 2), Ningxiner wine (*n* = 3), Zhuyeqing wine (*n* = 3), Jiafang wine (*n* = 2), Gucixiaotongye (*n* = 3), Guogong wine (*n* = 2), Huangjin wine (*n* = 1), Chinese Wolfberry wine (*n* = 1), were purchased or collected from various markets in China and stored at ambient temperature. Different Chinese medicinal raw materials are the main composition of them.

### Sample preparation

#### QuEChERS

The basic QuEChERS method with moderate modifications was used for extraction of target OCPs and PYPs from CM health wine samples. Initially, a carefully measured 5.0 mL sample of the CM health wine together with 5.0 mL of *n*-hexane: ethyl acetate (1:1, *v/v*) were added into the extractor. The mixture was then shaken vigorously for 2 min in order to induce phase separation and pesticide extraction. It was allowed to rest for a moment until obvious stratification had occurred. The bottom layer (organic phase) was removed and evaporated under a steam of N_2_ at 40 °C to a final volume of 1 mL. The concentrated solution was precisely measured by transferring it into a 1.0 mL volumetric flask. Next, the solution was placed in an eppendorf tube containing 400 mg florisil and shaken for 30 s using a vortex machine. Finally,, the sample was centrifuged at 12000 rpm for 5 min and the supernatant was filtered through a 0.22 μm filter for injection into the GC-ECD system.

#### SPE

Solid-phase extraction was performed using florisil SPE cartridges that had been previously conditioned with elutriant. First, 5.0 mL samples of diluted CM health wines were percolated through the cartridges at a constant flow rate. Next, the cartridges were rinsed with 20.0 mL of elutriant and vacuum-dried for 10 min. The pesticides that had been retained in the cartridges were then eluted with 2 × 5.0 mL of *n*-hexane:ethyl acetate mixture (1:1, *v/v*) and the eluate was collected in a test tube and concentrated to a 1.0 mL volume. Finally, the concentrated solution was filtered through a 0.22 μm filter prior to injection into the GC-ECD system for analysis.

## Additional Information

**How to cite this article**: Kong, W.-J. *et al.* Trace analysis of multi-class pesticide residues in Chinese medicinal health wines using gas chromatography with electron capture detection. *Sci. Rep.* **6**, 21558; doi: 10.1038/srep21558 (2016).

## Supplementary Material

Supplementary Information

## Figures and Tables

**Table 1:** Calibration data, LOD and LOQ, precision and stability of the GC-ECD method.

Pesticide	Calibration curve	*R*	Linear range(ng/mL)	LOD(ng/L)	LOQ(ng/L)	Precision (RSD, %)		Stability
						Intra-day	Inter-day	
Hexachlorobenzene	*y* = 267887*x*−1090	0.9994	0.001–1.0	0.03	0.1	1.37	1.17	0.56
Alpha-BHC	*y* = 486771*x*−5297.1	0.9988	0.001–1.0	0.02	0.06	1.46	1.42	0.45
Quintozene	*y* = 277789*x*−1979.6	0.9992	0.001–1.0	0.04	0.1	0.65	0.98	0.64
Gamma-BHC	*y* = 376449*x*−4154.8	0.9990	0.001–1.0	0.04	0.1	0.97	1.01	0.42
Heptachlor	*y* = 251364*x*−4725.5	0.9982	0.001–1.0	0.1	0.25	1.13	2.12	1.09
Aldrin	*y* = 316138*x*−977.87	0.9996	0.001–1.0	0.06	0.2	2.06	1.55	1.05
Chlorothalonil	*y* = 114350*x*−6606.8	0.9955	0.01–1.0	3.0	7.0	9.86	9.63	10.15
Beta-BHC	*y* = 134236*x*−1070.8	0.9993	0.001–1.0	0.8	0.3	3.18	2.63	2.74
Delta-BHC	*y* = 288462*x*−4851.4	0.9984	0.001–1.0	0.06	0.2	0.86	1.08	1.19
Heptachlor epoxide	*y* = 344132*x*−4890.8	0.9979	0.001–1.0	0.03	0.1	1.80	1.67	0.66
Triadimefon	*y* = 79059*x*−3567.6	0.9953	0.01–1.0	0.2	0.6	6.07	11.95	4.35
Alpha-endosulfan	*y* = 188142*x*−841.17	0.9998	0.001–1.0	0.06	0.2	1.18	1.92	0.18
Cis-chlordane	*y* = 65993*x*−1028.7	0.9944	0.005–1.0	0.06	0.2	1.51	1.31	0.50
Trans-chlordane	*y* = 69098*x*−423.5	0.9995	0.001–1.0	0.06	0.2	1.76	1.57	0.68
p.p′-DDE	*y* = 264411*x*−2860.7	0.9992	0.001–1.0	0.06	0.2	1.06	3.97	0.70
Dieldrin	*y* = 286981*x*−1528.9	0.9996	0.001–1.0	0.06	0.2	1.67	1.56	0.70
Endrin	*y* = 236659*x*−4419.4	0.9934	0.001–1.0	0.06	0.2	1.37	1.82	1.10
o.p′-DDT	*y* = 124345*x*−4164.4	0.9951	0.005–1.0	0.5	1.8	4.13	3.04	3.11
p.p′-DDD	*y* = 145814*x*−3592.2	0.9958	0.001–1.0	0.04	0.2	3.10	7.80	2.85
Beta-endosulfan	*y* = 108103*x*−455.58	0.9996	0.001–1.0	0.1	0.4	1.66	1.79	0.78
p.p′-DDT	*y* = 124664*x*−5688.9	0.9925	0.005–1.0	0.6	2	6.71	4.03	4.08
Methoxychlor	*y* = 32286*x*−1594.7	0.9947	0.001–1.0	1	4	5.58	7.88	2.94
Fenpropathrin	*y* = 76768*x*−3037.3	0.9947	0.01–2.0	0.06	0.2	5.73	12.00	5.17
Permethrin	*y* = 188912*x*−9252.3	0.9876	0.01–2.0	2	6	5.06	7.07	5.27
Cypermethrin	*y* = 11359*x*−799.08	0.9909	0.1–2.0	1	4	3.18	12.25	3.48
Flucythrinate	*y* = 31926*x*−2328.4	0.9960	0.1–2.0	0.4	1.5	3.65	13.18	4.41
Decamethrin	*y* = 19245*x*−1451.4	0.9918	0.1–2.0	1	3	2.39	8.82	3.02

*y*: peak area; *x*: concentration (ng/mL); *R*: correlation coefficient.

**Table 2:** Recoveries of 27 OCP and PYP pesticides in three kinds of fortified sample matrices.

Pesticide	Spikinglevel(mg/kg)	Recovery (*n* = 3)					
		Ningxiahong wine		Chinese Jing wine		Yedaolugui wine	
		Recovery(%)	RSD(%)	Recovery(%)	RSD(%)	Recovery(%)	RSD(%)
Hexachlorobenzene	0.005	64.00	10.18	57.80	1.86	60.82	3.77
	0.05	75.66	2.27	74.69	3.05	77.39	1.21
	0.5	94.74	3.28	89.54	2.50	90.72	3.64
Alpha-BHC	0.005	94.92	5.09	67.00	6.01	68.80	3.79
	0.05	80.38	2.32	79.63	1.54	82.18	0.93
	0.5	98.75	0.75	92.32	1.63	90.59	1.70
Quintozene	0.005	69.92	6.35	65.19	6.56	66.37	2.55
	0.05	82.99	2.73	77.63	0.99	74.86	0.51
	0.5	97.51	1.13	95.82	1.58	96.84	6.44
Gamma-BHC	0.005	69.19	5.51	67.81	9.12	64.04	7.89
	0.05	79.71	3.10	76.74	1.54	76.94	1.58
	0.5	96.82	0.75	87.19	1.82	87.83	1.75
Heptachlor	0.005	74.27	7.82	76.06	11.99	102.67	6.63
	0.05	86.11	5.76	96.39	4.79	87.57	1.00
	0.5	99.58	1.53	91.40	0.53	94.23	3.65
Aldrin	0.005	72.61	6.68	75.41	10.33	87.11	6.52
	0.05	74.96	2.95	78.97	1.43	79.05	0.52
	0.5	99.09	1.22	93.73	1.08	93.13	1.44
Chlorothalonil	0.005	50.38	5.79	64.69	5.87	110.21	11.48
	0.05	67.69	15.14	78.90	17.90	86.21	9.85
	0.5	120.31	2.88	113.65	2.54	105.03	1.01
Beta-BHC	0.005	90.86	5.97	93.08	1.47	95.76	4.64
	0.05	90.81	2.80	89.94	1.36	91.13	0.72
	0.5	101.27	2.84	94.09	3.43	97.13	1.14
Delta-BHC	0.005	69.64	4.00	75.89	3.46	77.94	3.58
	0.05	90.47	3.01	85.45	5.84	90.26	1.74
	0.5	103.23	0.80	95.15	1.70	95.88	1.67
Heptachlor epoxide	0.005	73.28	5.32	69.24	4.74	59.39	4.72
	0.05	82.73	2.37	78.79	1.00	89.02	1.10
	0.5	100.70	0.96	91.93	2.00	90.95	1.29
Triadimefon	0.005	62.41	14.02	62.96	10.26	53.83	7.14
	0.05	51.53	12.95	50.26	12.37	50.56	14.35
	0.5	62.96	1.34	47.74	6.13	52.71	0.36
Alpha-endosulfan	0.005	76.20	5.44	76.00	8.52	77.70	5.20
	0.05	78.91	2.34	84.23	1.30	83.26	0.73
	0.5	100.90	1.00	94.76	1.75	94.49	1.52
Cis-chlordane	0.005	77.01	5.02	78.06	6.96	78.61	9.48
	0.05	78.73	3.16	88.12	1.41	84.51	0.19
	0.5	101.27	0.87	96.32	1.44	95.20	1.03
Trans-chlordane	0.005	75.59	4.94	80.64	9.91	85.92	7.64
	0.05	77.39	2.65	84.67	1.28	84.08	0.90
	0.5	101.69	1.14	96.49	1.63	95.68	1.09
p.p′-DDE	0.005	71.71	8.20	71.60	9.50	78.17	6.34
	0.05	79.13	2.84	81.83	1.22	83.65	1.75
	0.5	104.13	1.55	99.52	0.67	98.81	1.63
Dieldrin	0.005	71.53	5.14	79.66	6.21	100.93	3.73
	0.05	82.29	3.18	98.65	1.49	101.91	5.37
	0.5	99.40	1.25	103.62	2.48	89.13	6.48
Endrin	0.005	76.48	5.52	81.44	5.20	94.09	3.94
	0.05	82.35	2.84	88.39	1.90	89.12	1.93
	0.5	100.93	1.31	94.58	1.84	95.20	1.81
o.p′-DDT	0.005	82.69	11.59	85.85	19.62	98.24	12.38
	0.05	84.44	14.54	90.20	15.63	94.74	6.16
	0.5	100.17	2.90	90.08	0.81	99.16	4.97
p.p′-DDD	0.005	77.65	5.60	83.69	3.65	88.25	6.26
	0.05	80.49	2.64	85.85	4.13	90.21	9.93
	0.5	106.33	1.36	101.86	2.80	101.68	0.48
Beta-endosulfan	0.005	71.96	6.57	79.80	8.20	107.61	5.05
	0.05	75.41	2.16	79.02	1.44	81.48	3.07
	0.5	90.02	2.11	80.31	1.67	85.74	1.00
p.p′-DDT	0.005	75.83	16.86	84.49	20.27	87.56	3.54
	0.05	96.82	24.69	97.80	23.56	107.49	9.75
	0.5	99.77	4.53	90.18	3.03	101.99	7.02
Methoxychlor	0.005	72.25	8.52	70.83	3.79	81.30	5.29
	0.05	84.78	15.48	86.35	13.40	93.02	3.00
	0.5	92.40	3.72	80.36	0.34	91.19	5.19
Fenpropathrin	0.01	95.27	4.12	94.61	4.45	88.66	4.55
	0.5	92.71	2.94	91.73	1.36	95.73	3.54
	1	100.09	2.17	91.87	1.46	98.24	2.55
Permethrin	0.01	97.19	11.99	97.40	2.34	105.97	7.24
	0.1	87.50	3.43	85.75	1.73	90.40	5.31
	1	103.65	3.09	97.19	0.88	102.76	3.37
Cypermethrin	0.01	98.00	5.52	97.60	3.44	96.66	4.89
	0.1	86.67	5.60	85.81	4.56	90.56	9.09
	1	101.61	4.27	92.50	1.96	102.17	5.79
Flucythrinate	0.01	85.45	4.74	98.19	1.11	86.97	9.19
	0.1	89.58	10.30	86.27	7.65	92.29	11.53
	1	114.70	4.28	104.70	2.69	107.53	8.09
Decamethrin	0.01	84.93	4.57	85.70	2.40	87.71	11.08
	0.1	85.56	13.05	82.66	10.10	96.43	16.09
	1	104.73	8.04	99.24	4.55	103.24	4.31

**Table 3:** Contents of 27 OCPs and PYP pesticides in 80 CM health wine samples.

SampleNo.	Name	Batch No.	Pesticides detected	Concentration(mg/L)
1	Ningxiahong wine	20051122 N1036	ND	ND
2	Ningxiahong wine	20091316 N2048	ND	ND
3	Ningxiahong wine	20091029 N1043	ND	ND
4	Ningxiahong wine	20090303 N1019	ND	ND
5	Ningxiahong wine	20081115 N1009	ND	ND
6	Ningxiahong wine	20090131 N1017	ND	ND
7	Ningxiahong wine	20100119 N1017	ND	ND
8	Ningxiahong wine	20090217 N1014	ND	ND
9	Ningxiahong wine	20090810 N1028	ND	ND
10	Ningxiahong wine	20090330 N1230	ND	ND
11	Ningxiahong wine	20100525 N1062	beta-BHC	31.55
12	Ningxihong wine	20090530 N2031	ND	ND
13	Ningxiahong wine	20090120 N2014	ND	ND
14	Ningxiahong wine	20090506 N2046	ND	ND
15	Ningxiahong wine	20071013 N2012	ND	ND
16	Ningxiahong wine	20090305 N1043	ND	ND
17	Ningxiahong wine	20091117 N2038	ND	ND
18	Ningxiahong wine	20090909 N2028	ND	ND
19	Ningxiahong wine	20100526 N2060	ND	ND
20	Ningxiahong wine	20090809 N2028	ND	ND
21	Chinese Jing wine	20100202/62	ND	ND
22	Chinese Jing wine	20100202/73	ND	ND
23	Chinese Jing wine	20090819/41	ND	ND
24	Chinese Jing wine	20100712/47	ND	ND
25	Chinese Jing wine	20100812/05	ND	ND
26	Chinese Jing wine	20100814/03	ND	ND
27	Chinese Jing wine	20100523/62	ND	ND
28	Chinese Jing wine	20100612/56	ND	ND
29	Chinese Jing wine	20100904/33	ND	ND
30	Chinese Jing wine	20100812/01	ND	ND
31	Chinese Jing wine	20101018/10	ND	ND
32	Chinese Jing wine	20100718/88	ND	ND
33	Chinese Jing wine	20100712/43	ND	ND
34	Chinese Jing wine	20100910/47	beta-BHC	5.67
35	Yedaolugui wine	20090824H	ND	ND
36	Yedaolugui wine	20100207	beta-BHC trans-chlordane	11.16 3.58
37	Yedaolugui wine	20091101H	ND	ND
38	Yedaolugui wine	20100112G	ND	ND
39	Yedaolugui wine	20100105H	ND	ND
40	Fenglin wine	LGG1210822	ND	ND
41	Fenglin wine	HFW1317670	ND	ND
42	Fenglin wine	FEY1613794	ND	ND
43	Fenglin wine	20091023/06	ND	ND
44	Diyi wine	20070608	ND	ND
45	Diyi wine	20100320	ND	ND
46	Diyi wine	20061224	ND	ND
47	Lotus white wine	20090321	beta-BHC	7.90
48	Lotus white wine	20090517	ND	ND
49	Lotus white wine	20090823	ND	ND
50	Lotus white wine	20100110	beta-BHC	7.11
51	Lotus white wine	20090420	ND	ND
52	Zhuyeqing wine	82621652	ND	ND
53	Zhuyeqing wine	201003011	ND	ND
54	Zhuyeqing wine	201004025	ND	ND
55	Yisheshengbao wine	20091008	beta-BHC	12.33
56	Yishebian wine	20090815	ND	ND
57	Guogong wine	9180080	ND	ND
58	Guogong wine	9180080	ND	ND
59	Shiguogong wine	100303	ND	ND
60	Jiafang wine	201005020	beta-BHC	11.75
			trans-chlordane	7.45
61	Jiafang wine	200909020	trans-chlordane	5.22
62	Huangjin wine	5.02E+12	ND	ND
63	Cordyceps Sinensis wine	20090512	ND	ND
64	Sanbian wine	20061213	ND	ND
65	Tezhisanbian wine	20090811033BJ	ND	ND
66	Ningxiner wine	20090404	ND	ND
67	Ningxiner wine	20090504	ND	ND
68	Ningxiner wine	20090620	ND	ND
69	Rhodiola rasea wine	Home made	ND	ND
70	Ginseng wine	Home made	alpha-BHC	2.48
			gamma-BHC	3.83
			beta-BHC	10.67
			delta-BHC	17.30
71	Lucid Ganoderma wine	Home made	ND	ND
72	Herba Saussureae Involucratae wine	Home made	ND	ND
73	Tall Gastrodia Tuber wine	Home made	ND	ND
74	Chinese Magnoliavine wine	Home made	ND	ND
75	Desertliving Cistanche Herb wine	Home made	ND	ND
76	Desertliving Cistanche Herb wine-2	Home made	ND	ND
77	Chinese Wolfberry wine	Home made	ND	ND
78	Gucixiaotongye wine	9180303	ND	ND
79	Gucixiaotongye wine	9180425	ND	ND
80	Gucixiaotongye wine	9181023	ND	ND

ND: not detected.
