# Supplementary material for: Trace analysis of multi-class pesticide residues in Chinese medicinal health wines using gas chromatography with electron capture detection
Source: Sci Rep. 2016 Feb 17;6:21558. doi: 10.1038/srep21558 (PMC4756289; doi:10.1038/srep21558)
Supplement: Supplementary Information [file srep21558-s1.doc]

**Trace analysis of multi-class pesticide residues in Chinese medicinal health wines using gas chromatography with electron capture detection**

Wei-Jun Kong, Qiu-Tao Liu, Dan-Dan Kong, Qian-Zhen Liu,

Xin-Ping Ma & Mei-Hua Yang*

Table 1. Basic parameters for the characterization of separation profile of target pesticides under the optimized GC-ECD conditions.

| Pesticide | Resolution | TPN^a^ | Pesticide | Resolution | TPN^a^ |
| --- | --- | --- | --- | --- | --- |
| Hexachlorobenzene | 8.90 | 217226 | p.p′-DDE | 6.11 | 562580 |
| Alpha-BHC | 18.75 | 320294 | Dieldrin | 8.3 | 626845 |
| Quintozene | 5.62 | 296718 | Endrin | 4.45 | 684823 |
| Gamma-BHC | 12.79 | 285463 | o.p′-DDT | 14.3 | 875717 |
| Heptachlor | 8.62 | 247455 | p.p′-DDD | 1.88 | 775588 |
| Aldrin | 11.43 | 212351 | Beta-endosulfan | 4.64 | 972648 |
| Chlorothalonil | 11.11 | 265309 | p.p′-DDT | 25.91 | 730816 |
| Beta-BHC | 3.41 | 303298 | Methoxychlor | 2.81 | 589008 |
| Delta-BHC | 6.54 | 381429 | Fenpropathrin | 22.75 | 299762 |
| Heptachlor epoxide | 4.81 | 386184 | Permethrin | 4.17 | 283160 |
| Triadimefon | 2.56 | 360815 | Cypermethrin | 20.82 | 578680 |
| Alpha-endosulfan | 2.6 | 447071 | Flucythrinate | 12.46 | 396237 |
| Cis-chlordane | 3.23 | 494722 | Decamethrin | 15.07 | 268683 |
| Trans-chlordane | 3.72 | 550491 |  |  |  |

^a^ Theoretical plate number.


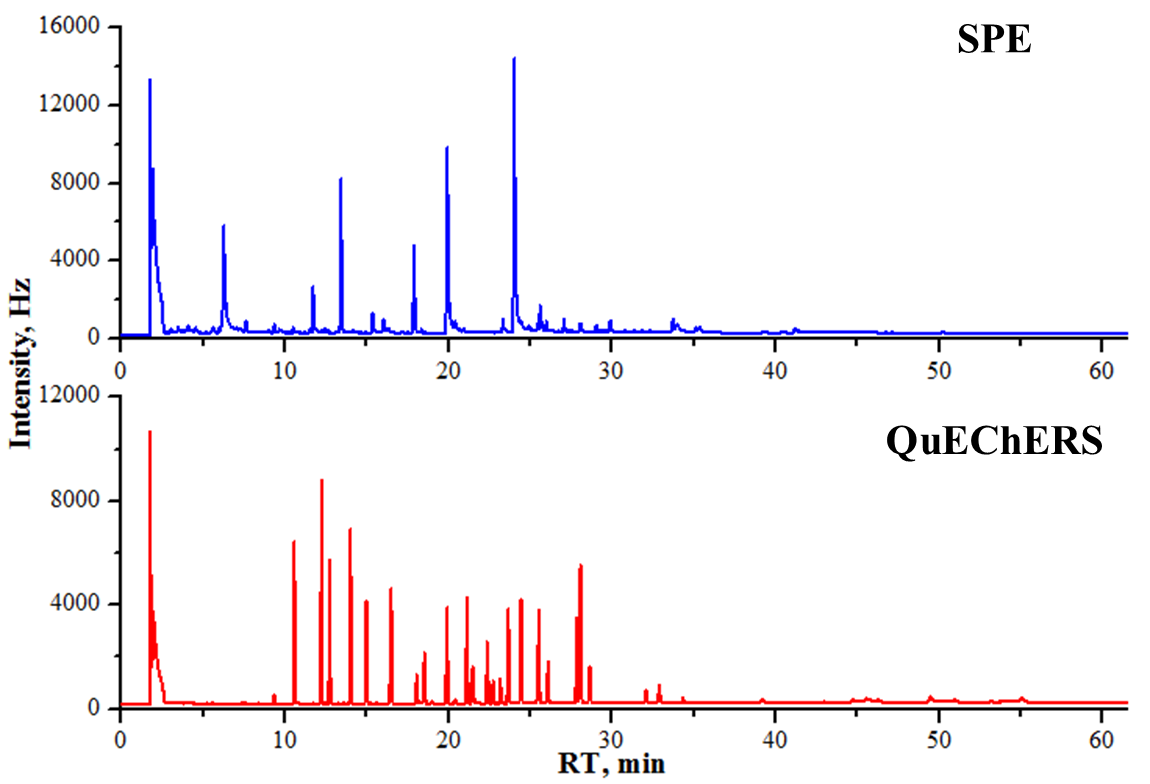


Figure 1. Comparison of SPE and QuEChERS based method for sample preparation.
